# TOL19-001 reduces inflammation and MMP expression in monolayer cultures of tendon cells

**DOI:** 10.1186/s12906-015-0748-7

**Published:** 2015-07-09

**Authors:** Catherine Baugé, Sylvain Leclercq, Thierry Conrozier, Karim Boumediene

**Affiliations:** Normandie Univ, Caen, France; EA4652 Equipe BioConnecT, UFR de médecine, Université de Caen, CS14032 Caen cedex 5, Caen, France; Clinique Saint-Martin, Caen, France; Service de rhumatologie, Hôpital Nord Franche-Comté, Belfort, France

**Keywords:** Cytokines, Tendons, Tendinopathy, Spirulina, Quinolone, Ciprofloxacin, Interleukin-1, Matrix metalloproteinases, Inflammation, Dietary supplements, Humans, *In vitro* model

## Abstract

**Background:**

Tendinopathies are tendon conditions associated with degeneration and disorganization of the matrix collagen fibers, tendon cells apoptosis and inflammation through up-regulation of proinflammatory cytokines, matrix metalloproteinase (MMP) expression, and prostaglandin E_2_ (PGE_2_) production. Currently, the pharmacological treatment is mainly based on non-steroidal anti-inflammatory drugs (NSAIDs) use and corticosteroid injections, which both can lead to numerous side effects for patients. TOL19-001 is a diet supplementary composed mostly of spirulina and glucosamine sulfate whose antioxidant properties could be helpful to treat tendinopathies while avoiding taking NSAIDs. In this study we developed an *in vitro* model of tendinopathy in order to evaluate the therapeutic potential of TOL19-001.

**Methods:**

Tendon cells were cultured on monolayer and treated with interleukin-1β (IL-1β) or ciprofloxacin (CIP), and then, MMPs, PGE2 and collagen expression was evaluated by RT-PCR or Elisa. In addition, a cotreatment with increased doses of TOL19-001 was done. Toxicity of TOL19-001 was evaluated using a metabolic activity assay.

**Results:**

This study demonstrates that IL-1β mimics some aspects of tendinopathies with PGE2 induction, MMP expression (mostly MMP1 and MMP3), and increases of type III/I collagen ratio. CIP, meanwhile, leads to an increase of MMP2 and p65 mRNA, whereas it reduces TIMP1 expression. Scleraxis expression was also increased by CIP whereas it was decreased by IL-1β treatment. Besides, TOL19-001 cotreatment suppresses tendon cell inflammation *in vitro*, marked by the downregulation of PGE2, MMPs and type III collagen in IL-1β stimulated-cells. TOL19-001 also represses CIP induced-changes.

**Conclusions:**

These findings indicate that TOL19-001 exerts anti-inflammatory effects on tendon cells, which might explain why TOL19-001 diet may improve tendon function in patients with tendon injury. Future research is required to determine TOL19-001 effect on injured or overused tendons in vivo.

## Background

A tendon is a fibrous connective tissue which attaches muscle to bone, and transmits force producing movements. Tendons also function to stabilize joints and absorb large shocks, protecting muscles from damage. The major elements of the extracellular matrix are collagen fibers which represent about 65 to 80 % dry weight of tendon. These collagen fibers, which are composed of type I collagen (95 % of collagens) [1], and of some minor collagens (collagen III, V and X), provide the tendons with strength to withstand high loads. Proteoglycans, such as decorin, glycoproteins and elastin also composed tendon matrix [1, 2]. These specific matrix components give tendon its resilience and biomechanical stability.

The cellular component is represented by tenoblasts and tenocytes, which are arranged in parallel rows between the collagen fibers. Tenoblasts are immature spindle-shaped tendon cells, containing abundant cytoplasmic organelles, reflecting their high metabolic activity. As they age, tenoblasts become elongated and transform into tenocytes. Together, tenoblasts and tenocytes account for 90 to 95 % of the cellular elements of tendons. The remaining cellular elements consist of chondrocytes, synovial cells and endothelial cells [3]. Tenocytes have a low mitotic activity and are poorly vascularized. Consequently, the metabolic rate of tendons is relatively limited; oxygen consumption is 7.5 times lower than that of skeletal muscle and the turnover time for tendon collagen varies from 50 to 100 days [4]. So, damaged tendons are difficult to regenerate [5], and its recovery after injury takes time.

Historically, chronic pain referring to a symptomatic tendon was called “tendinitis”, implying inflammation as a central pathological process. The more generically term “tendinopathy” (TP) is now currently preferred [3, 6]. These tendon disorders are common and account for a high proportion of referrals to rheumatologists and orthopedic surgeons [7]. Several factors have been implicated in TP pathogenesis, most of which may cause localized inflammatory reactions and also microdegeneration. Genetic background and age may also play a role. Additionally, the use of several drugs has been associated with TPs: the association has been proven for fluroquinolone antibiotics [8], whereas the responsibility of statins [9], oral contraceptives and locally injected corticosteroids [10, 11] is still debated.

Tendon healing occurs in three distinct but partially overlapping phases [12]. The acute inflammatory phase lasts for up to 3 to 7 days after injury. During this phase, inflammatory agents such as interleukin-1β (IL-1β) are produced by macrophages and other inflammatory cells at the injured site. Then, the proliferation phase lasts between 5 and 21 days. Tenocytes produce collagen, which gradually increases the mechanical strength of the tendon, so that loading can lead to elastic deformation. The last phase is the maturation and remodeling phase and it can take place for up to a year. The cross-linking among collagen fibers increases and the tensile strength, elasticity and structure of the tendon are improved.

The healing process [13] is primarily mediated by matrix metalloproteinases (MMPs) and metalloproteinases with thrombospondin motifs (ADAMTs) [14] and their tissue inhibitors (TIMPs) [15]. These enzymes participate in both collagen degradation and remodeling [14]. In addition, during TP, changes in rates of collagen were observed, including an increase in the proportion of collagen type III compared to collagen type I. Wounding and inflammation also provoke the release of growth factors and cytokines from platelets, polymorphonuclear leukocytes, macrophages and other inflammatory cells. These growth factors induce neovascularization and stimulate fibroblasts and tenocyte proliferation and synthesis of collagen [16]. PGE2 could also be involved in the healing process. PGE2 is a potent inhibitor of type I collagen synthesis [17–19] and it has recently been shown that PGE2 has catabolic effects on tendon structure, decreasing proliferation and collagen production in human patellar tendon fibroblasts [20].

Since tendon injuries are common in adults, and require more than 300,000 surgeries of tendons each year in the U.S. [21], it is important to identify substances that can improve the treatment or prevented tendon injuries. Non-steroidal anti-inflammatory drugs and corticosteroids are commonly prescribed to minimize inflammation and subsequent damage to tissue integrity [22]. They may be beneficial for pain and function in the early phases of disease, but are usually ineffective later [23, 24], and associated with numerous side effects including inhibitory effects on proteoglycan synthesis and cell proliferation [25]. Therefore, investigation continues for safer and more selective pharmaco-therapies for tendinopathies. Preliminary studies utilizing Platelet-Rich-Plasma (PRP) [26], anakinra (an interleukin-1 antagonist) [26] or apronitin (a MMP-inhibitor) [28] have produced encouraging results, suggesting that substances targeting IL-1β or inhibiting MMPs may be useful. Here, we propose that the association of spirulina, glucosamine sulfate, ginseng, selenium, sillicium, iron, vitamin E and zinc (TOL19-001, marketed as Cicatendon®, LABRHA Laboratory, Lyon, France) may have a beneficial effect on tendon healing and repair.

In this study, we developed two original models to investigate *in vitro* effects of drugs on a heterogeneous population of human resident tendon cells in adherent monolayer culture, and showed that TOL19-001 efficiency reduces inflammation and MMP expression.

## Methods

### Reagents

Reagents were supplied by Invitrogen (Fisher Bioblock Scientific, Illkirch, France) unless otherwise noted. IL-1β (Sigma-Aldrich, St. Quentin Fallavier, France) was resuspended in phosphate buffered saline (PBS) with BSA. Ciprofloxacin (CIP) was provided by Fresenius Kabi (Sèvres, France). Oligonucleotides were supplied by Eurogentec (Angers, France). TOL19-001 (Cicatendon®, Labhra, Lyon, France) was resuspended in PBS.

### Tendon cell culture

Tendons from the pyramidal muscle were obtained from patients undergoing arthroscopic surgery. All donors signed agreement forms before surgery, according to local legislations (agreement #A13-D46-VOL.19 obtained from local ethical committee, “Comité de Protection des Personnes Nord Ouest III”). Human resident tendon cells were isolated by washing several times tendon samples with phosphate buffered saline (PBS), dissection into small pieces and incubation with 2 % collagenase type II in DMEM supplemented with 10 % fetal calf serum (FCS) overnight at 37 °C. The digested material was then placed in a tissue culture flask and cultured in DMEM supplemented with 10 % FCS and antibiotics. Cells were incubated at 37 °C, 95 % humidity and 5 % carbon dioxide with a change of medium every 2–3 days.

### Experimental design

In all experiments, tendon cells after one passage were used to prevent their dedifferentiation.

Tendon cells were treated at confluency with IL-1β or CIP in the presence or not of TOL19-001 for 48 h. Then, different analysis were performed on cells or culture medium.

### RNA Extraction and real-time reverse transcription–Polymerase chain reaction

Total RNA from primary tendon cell cultures were extracted using Trizol® Reagent (Life Technologies, #15596-018) [27, 28]. After extraction, 1 μg of DNase-I–treated RNA was reverse transcribed into cDNA in the presence of oligodT and Moloney murine leukemia virus reverse transcriptase. The reaction was carried out at 37 °C for 1 h followed by a further 10-min step at 95 °C. Amplification of the generated cDNA was performed by real-time PCR in an Applied Biosystems SDS7000 apparatus with appropriate primers designed with Primer Express software. The relative mRNA level was calculated with the 2^–ΔΔCT^ method.

### WST1 assay

Cytotoxicity was evaluated by tetrazolium colorimetric WST1 assay (Product No 05015944001, Roche Diagnostics, Meylan, France) [29, 30]. Tetrazolium salts are cleaved to formazan by the succinate-tetrazolium reductase system (EC 1.3.99.1) which belongs to the respiratory chain of the mitochondria, and is only active in metabolically intact cells. Therefore, the amount of formazan dye formed (which can be quantitated with a scanning multi-well spectrophotometer) directly correlates to the number of metabolically active cells in the culture.

Tendon cells (undergoing one passage) were plated into 96-well microtiter plates. At 70 % confluence, cells were incubated with CIP (100 μg/ml) or IL-1β (1 ng/ml). One hour before the end of incubation, 10 μl of WST1 solution were added to all wells. Optical density was measured on a spectrophotometer plate reader (1420 Multilabel Counter, Perkin Elmer) at 450 nm. A well without cells containing complete medium and WST1 only acted as blanks.

### Elisa

PGE2 and MMPs released into conditioned media was quantified using commercially available enzyme immunoassay kit (R&D Systems). Absorbance was determined at 450 nm with a wavelength correction set at 540 nm.

### Statistical analysis

Three different experiments were performed. The values are means ± standard deviation and the significance of differences was calculated with Student’s *t* test.

## Results

This study was undertaken to investigate the effect of TOL19-001® in human tendon cells in two *in vitro* models of tendinopathies. However, such models do not currently exist. Since tendon overuse injuries and tendinopathies are accompanied by inflammation through up-regulation of proinflammatory cytokines, matrix metalloproteinase (MMP) expression, and prostaglandin E_2_ (PGE_2_) production, which could initiate the degradation and remodeling of tendons [31, 32], we tried to reproduce these changes in tendon cell culture. Two approaches were tested: the first one was a treatment with a fluoroquinolone antibiotic, called ciprofloxacin (CIP); the second one consists to treat cells with a proinflammatory cytokine, IL-1β.

### CIP and IL-1β differently induced MMP expression in tendon cells

**Fig. 1:**
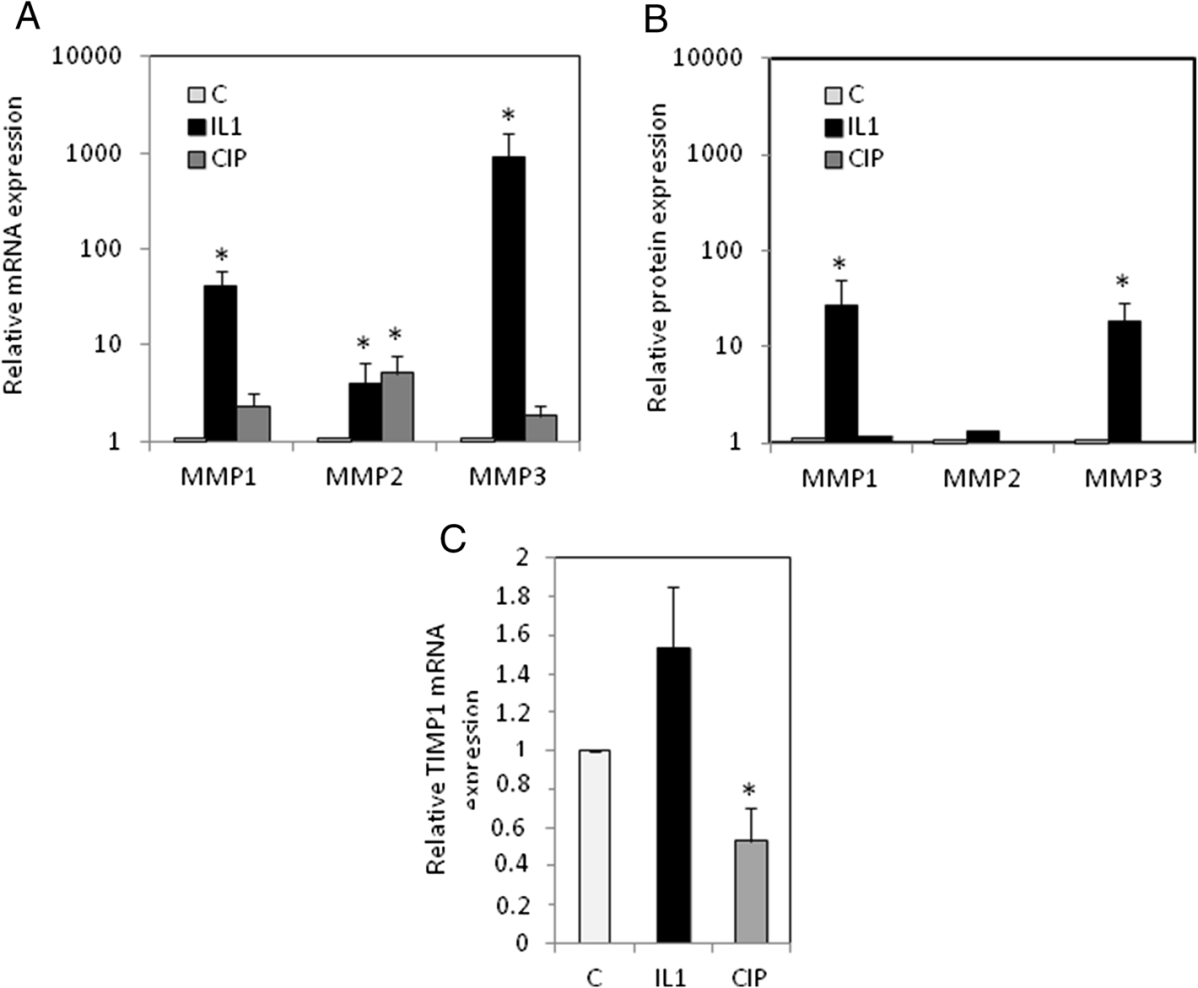
Effect of CIP and IL-1β on MMPs and TIMP-1 expression. Tendon cells (have been incubated with CIP (100 μg/ml) or IL-1β (1 ng/ml) for 48 h. Next, MMP1, MMP2 and MMP3 mRNA levels were determined by RT-PCR (**a**), and MMP release by ELISA (**b**). TIMP1 mRNA expression was also determined by RT-PCR (**c**). Histograms represent mean values from 3 independent experiments ± SEM

First, we analyzed the induction of MMPs (MMP1, 2, 3) by CIP and IL-1β (Figure 1). Tendon cells (at passage 1) were treated with IL-1β (1 ng/ml) or CIP (100 μg/ml) for 48 h. By RT-PCR and Elisa, we found that IL-1β strongly increased MMP1, MMP2 and MMP3 mRNA level, and release in medium. The effect on MMP2 was more moderated compared to MMP1 and MMP3. CIP, meanwhile, induced a slight increase of MMP1, MMP2 and MMP3 mRNA levels. The induction of MMP2 was more important compared to other MMPs, and similar to this obtained with IL-1β treatment. However, no significant effect on MMP release in medium was observed after CIP treatment.

Furthermore, we investigated the effect of IL-1β and CIP on TIMP1 expression, and showed that 48 h-treatment with IL-1β increased TIMP1 mRNA level, whereas CIP reduced it.

### CIP, but not IL-1β increased p65 mRNA expression

**Fig. 2:**
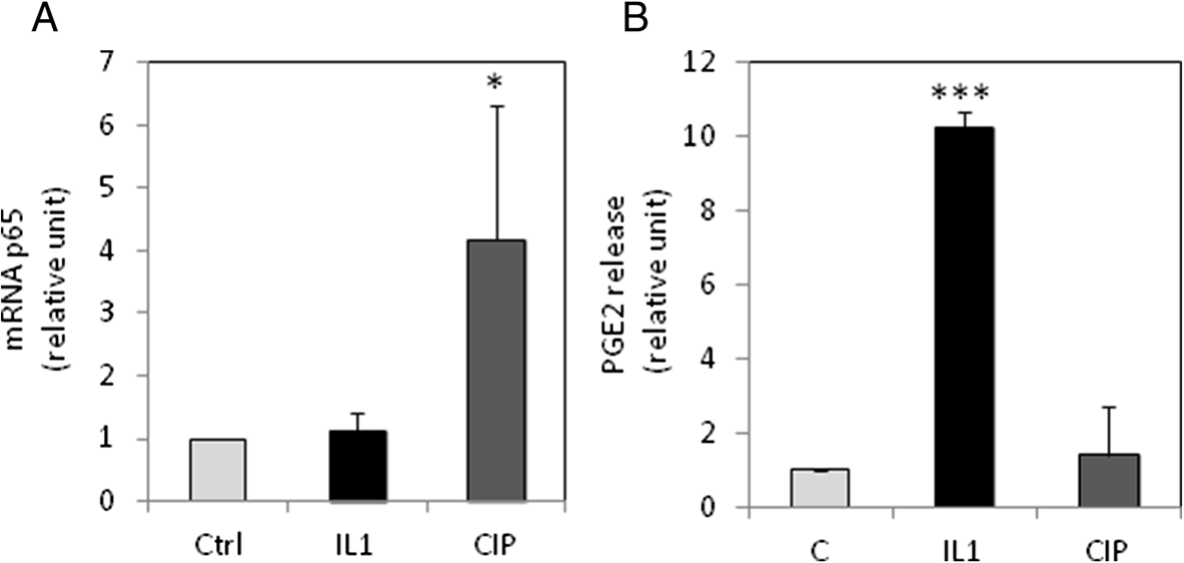
Effect of CIP and IL-1β on p65 mRNA level and PGE2 release. Tendon cells were treated as Fig. 1, and p65 mRNA level (**a**) and PGE2 release (**b**) were determined by RT-PCR and ELISA, respectively. Histograms represent mean values from 3 independent experiments ± SEM

Since it is known that MMPs are regulated, in part, by NFκB pathway, we hypothesized that IL-1β and CIP may activate this signaling pathway in tendons cells. Insofar as we have previously observed that IL-1β regulates NFκB signaling in chondrocytes, in part, by increasing mRNA expression of the NFκB subunit p65, we postulated that, in tendon cells, IL-1β and CIP may also upregulate p65. Surprisingly, IL-1β (1 ng/ml) did not induce an increased p65 mRNA level in tendon cells after 48 h-treatment (Fig. 2a). However, CIP increased its expression. This suggests that CIP and IL-1β have different mechanisms of action.

### IL-1β, but not CIP, induced PGE2 in tendon cells

We also investigated the effect of IL-1β and CIP treatment on PGE2 expression (Fig. 2b). As for MMPs, we found a differential response of tendon cells to treatments. In our culture conditions, only IL-1β was able to significantly induce PGE2 release by tendon cells.

### IL-1β downregulated type I collagen and scleraxis, whereas it upregulated type III collagen

**Fig. 3:**
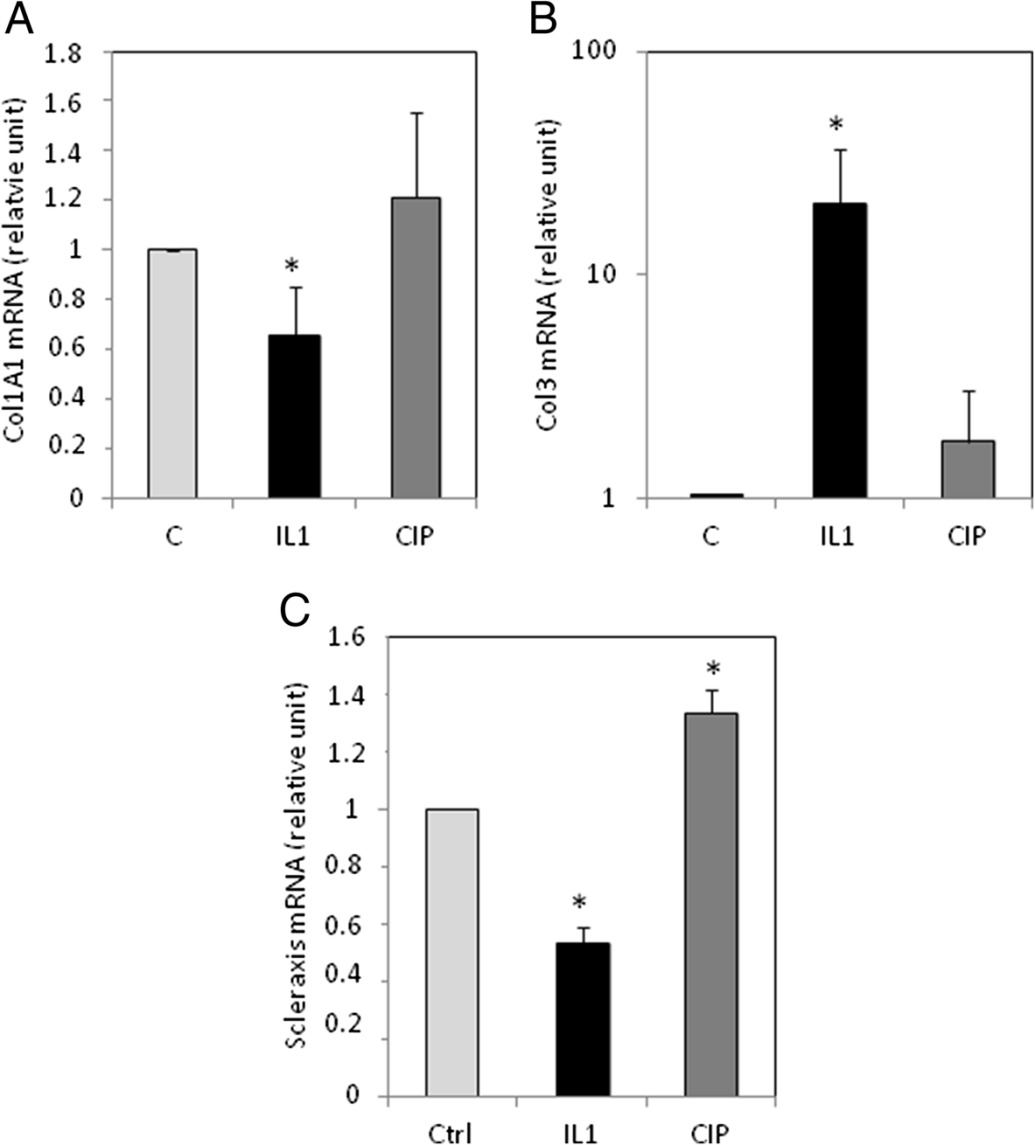
Effect of CIP and IL-1β on type I and III collagen and scleraxis mRNA level. Tendon cells were treated as Fig. 1, and type I and III collagen (**a**, **b**) and scleraxis (**c**) mRNA expression was evaluated by RT-PCR. Histograms represent mean values from 3 independent experiments ± SEM

Furthermore, we examined whether CIP or IL-1β treatment modify matrix gene expression (Fig. 3a and b). We focused on type I and type III collagens, the major components of tendon matrix. Interestingly, we found that IL-1β treatment reduced type I collagen mRNA level, whereas it increased type III collagen expression. In contrast, we could not find a significant effect of CIP.

It is known that type I collagen is regulated in part by scleraxis in tenocytes. Therefore, we investigated the effect of IL-1β on scleraxis expression (Fig. 3c), and showed that IL-1β down-regulated scleraxis mRNA that could explained the reduction of type I collagen by IL-1β.

### TOL19-001® is not toxic for tendon cells

**Fig. 4:**
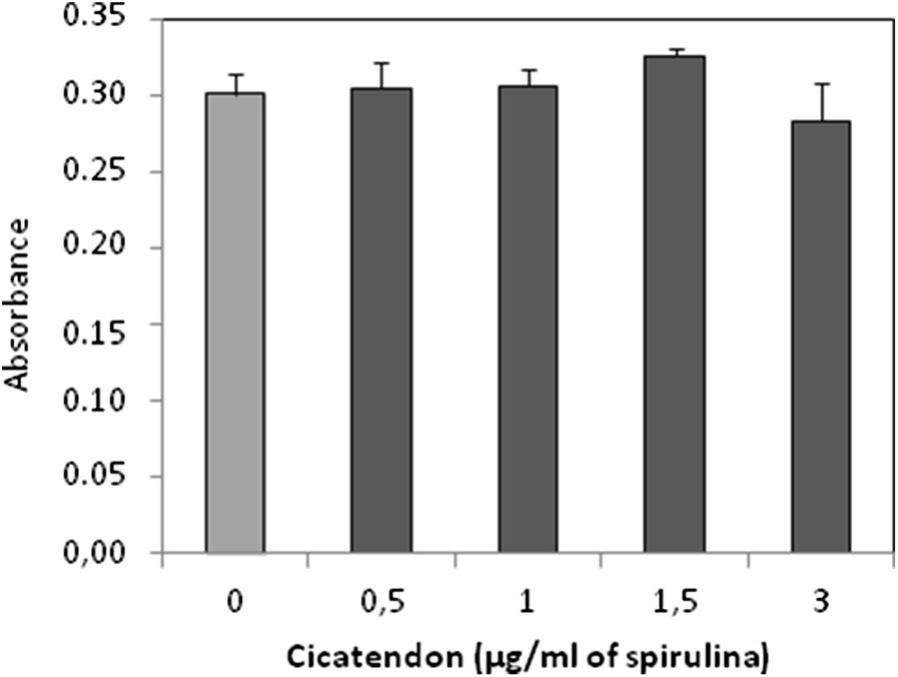
Effect of TOL19-001® on metabolic activity of tendon cells. Tendon cells (at passage 1) have been incubated in the presence of TOL19-001® (dose equivalent to 0.5 to 3 μg/ml of spirulina) for 48 h. Then, metabolic activity was evaluated by Wst1 assay. Values were normalized to OD from untreated cells. Histograms represent mean values from 3 independent experiments ± SEM

Tendon cells treated with TOL19-001 (0.5 – 3 μg/ml of spirulina) showed no sign of cytotoxic effects or any negative effects on cell viability (data not shown) at the light microscopic. In addition, treatment did not affect cell proliferation as shown by Wst1 assay (Fig. 4).

### TOL19-001® reduced PGE2 and MMP expression in IL-1β stimulated- tendon cells

**Fig. 5:**
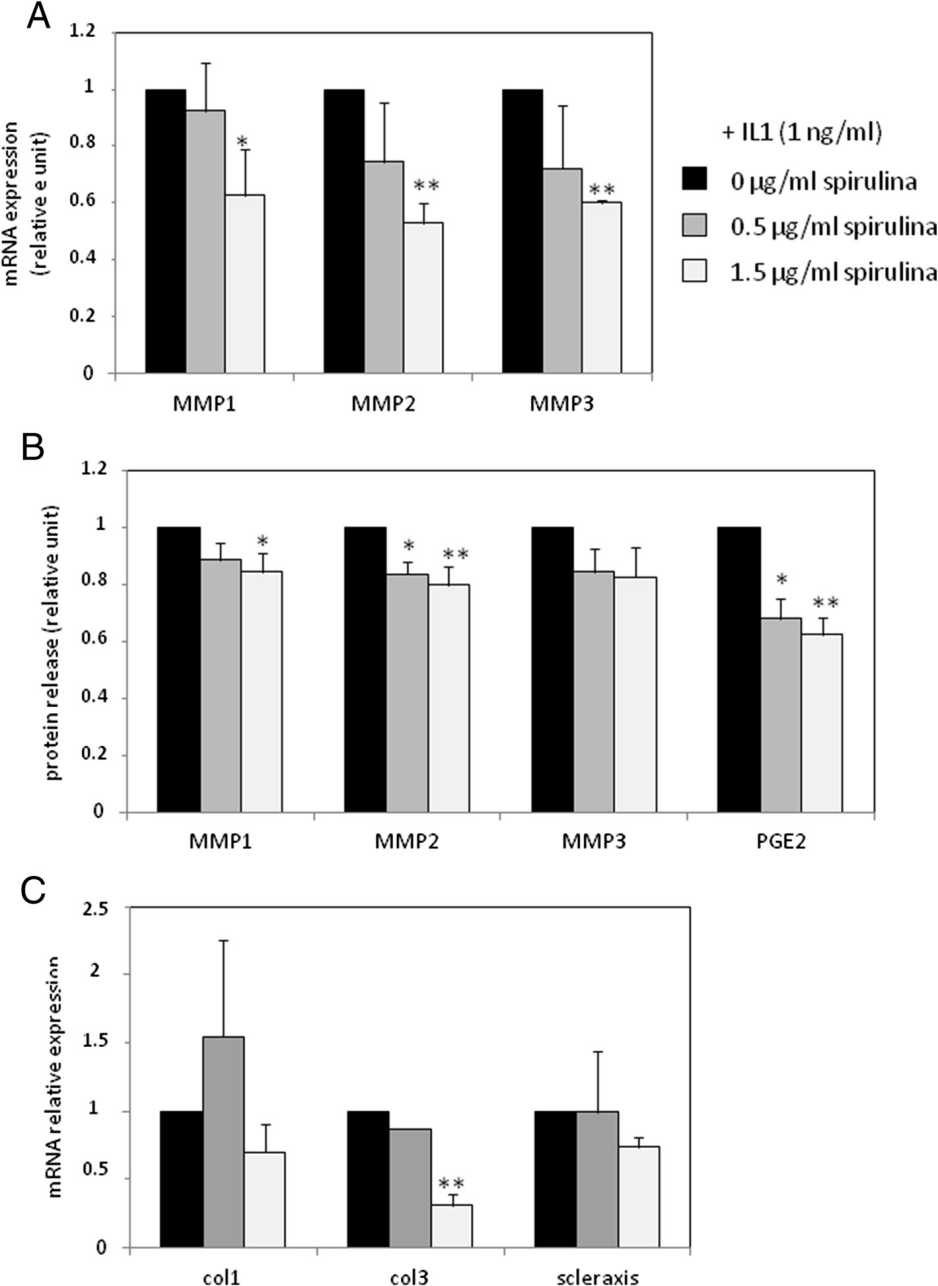
Effect of TOL19-001® in IL-1β stimulated-tendon cells. Tendon cells have been treated with IL-1β (1 ng/ml) and TOL19-001 (dose equivalent to 0.5 μg/ml and 1.5 μg/ml of Spirulina) for 48 h. Then, MMP, PGE2, collagen and scleraxis expression was evaluated by RT-PCR (**a** and **c**) or ELISA (**b**). Histograms represent mean values from 3 independent experiments ± SEM

Co-treatment with TOL19-001 reduced the effects of IL-1β. Indeed, at mRNA level, TOL19-001, (used at 1.5 μg/ml of spirulina), significantly inhibited the induction of MMP1, MMP2 and MMP3 (by about 40 %) (Fig. 5a). These effects were also found when we analyzed MMP release in medium, but were less important (Fig. 5b). In addition, TOL19-001 impaired PGE2 release in IL-1β stimulated-tendon cells (Fig. 5B). Interestingly, co-treatment of tendon cells with TOL19-001 also reduces type III collagen expression in IL-1β treated-cells (Fig. 5c).

### TOL19-001® decreased MMP2 and p65 expression in CIP stimulated-tendon cells

**Fig. 6:**
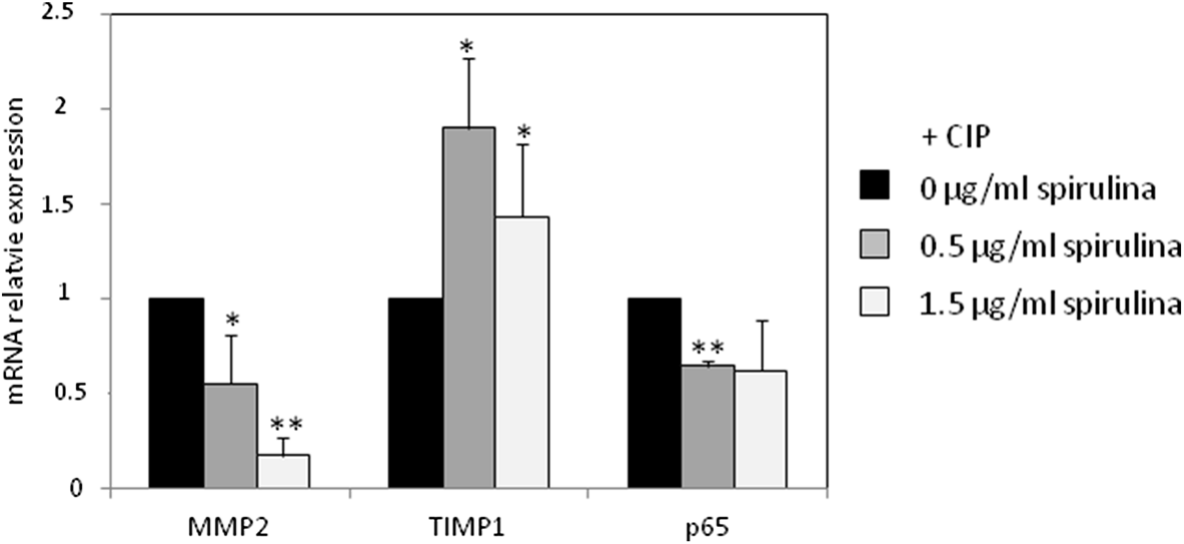
Effect of TOL19-001® in CIP stimulated-tendon cells. Tendon cells have been treated with CIP (100 μg/ml) and TOL19-001 (dose equivalent to 0.5 μg/ml and 1.5 μg/ml of Spirulina) for 48 h. Then, MMP2, TIMP1 and p65 mRNA expression was evaluated by RT-PCR. Histograms represent mean values from 3 independent experiments ± SEM

We, also, examined whether TOL19-001 was also able to counteract CIP effects on tendon cells. TOL19-001 co-treatment reduced MMP2 and p65 mRNA expression, whereas it increased that of TIMP1 in CIP stimulated-tendon cells (Fig. 6).

These results showed that TOL19-001 counteracts most of CIP and IL-1β induced-effects on tendon cells.

## Discussion

A major difficulty to identify efficient treatments against TPs is the absence of models easily usable in laboratory. Indeed, there is currently no *in vitro* nor in vivo model capable of mimicking all the conditions of these pathologies. Here, we used two different condition cultures able to induce some modifications comparable to human TP pathologies, which are accompanied by inflammation and degradation of the tendon extracellular matrix.

The first model was a treatment of monolayer culture of tendon cells with CIP, a fluoroquinolone antibiotic. This family of drugs is, indeed, known to induce tendon lesions in vivo [33–40] by causing matrix disruption, inflammation, and degenerative changes of tenocytes [37, 41]. In this study, we showed that CIP affects tendon cells, including inhibition of cell proliferation (data not shown), increased expression of p65 NFkB subunits and MMPs (at least at mRNA level). However, unlike others works [42, 43], we did not observe a reduction of matrix genes.

The second model consisted to treat monolayer cell cultures with IL-1β [44]. Indeed, pro-inflammatory cytokines such as IL-1β have been identified as the main initiators of tendinopathy, stimulating inflammation, apoptosis and matrix degradation [45]. At a tendon injury site, pro-inflammatory cytokines such as IL-1β may initiate a cascade of events leading to tendon destruction and loss of biomechanical structural integrity. Furthermore, besides the up-regulation of inflammatory mediators (PGE2), we found that IL-1β significantly up-regulates MMPs, and down-regulates the expression of type I collagen whereas it increases type III collagen mRNA in tendon cells. These modifications of matrix gene expression are coherent with changes of tissue remodeling observed during TPs. Indeed, whereas normal tendons mainly comprise type I collagen, injured tendons have a higher percentage of type III collagen, which is deficient in the number of cross-links between and within the tropocollagen units [46]. Surprisingly, we did not observe an increased expression of p65 mRNA after IL-1β treatment. However, it is possible that the IL-1β dose or treatment time was inadequate to observe NFkB activation. Indeed, it is well known that IL-1β acts very quickly on NFkB pathway (about minutes). However, here, we analysed effects of IL-1β on tendon cells after 48 h-treatments. It may be too long [47]. Beside, here, we used a single concentration of IL-1β (1 ng/ml) whereas in the majority of previous study showing NFkB activation in tenocytes, IL-1β was used at higher concentration [48–50]. So, we may observe an effect whether we had studied higher dose of IL-1β, or used a shorter incubation time. In addition, we could not exclude that, in tenocytes, IL-1β induces NFκB activation through a post-transcriptional regulation. Indeed, NF-κB is present in the cytoplasm in its resting stage as a heterotrimer complex consisting of two subunits and an additional inhibitory subunit, IκBα [51]. During the activation process, the inhibitory subunit IκBα is phosphorylated at Ser-32 and Ser-36 residues by IKK kinase (IκBα kinase) and is subsequently degraded. Once released, subunits of activated NF-κB translocate to the nucleus and mediate transcription of various inflammatory and catabolic gene products [52, 53]. Thus, it is likely that IL-1β induces NF-κB activation in tenocytes [44], whereas it does not upregulate p65 mRNA expression, and consequently by a different mechanism than CIP. IL-1β may also act through the activation of numerous other signal transduction systems such as MAP kinases.

Although CIP and IL-1β have been several times used in tenocytes [42, 43, 54, 55], this is, at our knowledge, the first report comparing their effects. This work shows that CIP has moderate effects on tendon cells, compared to IL-1β, suggesting that IL-1β treatment is a better model to mimic tendinopathies *in vitro* (with an increase of MMPs, PGE2 and type III collagen expression) than CIP. However, a CIP treatment may also be used to mimic fluroquinone-induced TP.

Next, we used these both treatments to investigate the effect of a nutraceutical formulation composed mainly of *Spirulina* (TOL19-001®) on tendon cells. We found that TOL19-001® reduces the expression of MMPs, PGE2 and type III collagen in IL-1β stimulated cells. In addition, in CIP-treated cells, TOL19-001® decreases MMP2 and p65 mRNA expression, whereas it increases one of TIMP1. This data suggest that TOL19-001® may be benefit for patients suffering of tendinopathies.

The downregulation of PGE2, which is present at high levels in injured tendons and causes vasodilatation [56] and hyperalgesia [57], may reduce pain associated with tendon inflammation. Indeed, PGE2 can increase the amount of substance P, a major neuro-transmitter of pain sensations which is released in sensory nerves [58].

Furthermore, healing tendons eventually form type III collagen-rich scar tissue, which impairs tendon function and makes the tendon susceptible to re-injury because the scar tissue has inferior mechanical properties compared to intact tendons (rich in type I collagen) [59]. In these conditions, TOL19-001® may have a benefit effect on healing in reducing type III collagen production.

Besides, TOL19-001® reduces expression of MMPs, which may reduce the deterioration of tendons. Indeed, matrix metalloproteinases are known to be involved in tendon matrix degradation [60, 61] due to their ability to degrade various ECM components, including collagen, fibronectin, and proteoglycans [62]. MMPs are expressed in normal tendon and upregulated during tendon healing [63, 64]. Given their role in the degradation and remodeling of tissue, we hypothesize that the inhibition of MMP expression induced by TOL19-001® might favor regenerative processes and tendon scarring.

TOL19-001® is mainly composed of Spirulina (*S. maxima*) (about 60 %), glucosamine sulfate (~20 %), and ginseng (~15 %). Consequently, it is difficult to know which component is responsive for TOL19-001® action. *Spirulina (Arthrospira) maxima*, is an undifferentiated multicellular filamentous cyanobacterium (blue-green alga) [65] that has a long history of use as food. Early interest in Spirulina focused mainly on its potential as a source of protein and vitamins [65, 66], but recently more attention has been made to study its therapeutic use, and a number of published reports suggest beneficial effects of this microalgae. Spirulina have anti-cancer and immune suppressing actions [67–69], antioxidant properties [70] and anti-inflammatory activity [71–73]. Antioxidant properties of this cyanobacterium are attributed to molecules, such as c-phycocyanin, β-carotene, tocopherol, γ-linolenic acid, and phenolic compounds. The c-phycocyanin from *Spirulina* has been shown to exert strong free radical scavenging activity [74]. Studies *in vitro* and in vivo have shown that the antioxidant components produced by *Spirulina* [65, 75] can prevent or delay oxidative damage by reducing the accumulation of ROS [76] through the activation of the antioxidant enzyme systems of catalase (CAT), superoxide dismutase (SOD), and glutathione peroxidase (GPx) [77]. Besides, the anti-inflammatory activity of phycocyanin has been demonstrated in various *in vitro* studies and in vivo experimental models such as mice with arthritis or sepsis [78–80]. In addition to Spirulina, glucosamine sulfate may also be responsive for anti-inflammatory effects of TOL19-001® in tendon cells.

## Conclusions

Tendon injuries are a common condition encountered in orthopaedic surgery and sport medicine practice. In the past few decades, incidences of tendon injuries have increased significantly in both recreational and highly-competitive athletes. NSAID are insufficient for treat tendinopathies due to their numerous side effects. Here, thanks to the characterization of *in vitro* models using tendon cells cultured in monolayer to study TP, we show that diet supplementary enriched in Spirulina and glucosamine sulfate, such as TOL19-001®, may be benefic against TP by regulating inflammation and tendon matrix remodeling.
